# Special Issue: Formation and Function of Fungal Ascospores

**DOI:** 10.3390/jof7080618

**Published:** 2021-07-29

**Authors:** Aaron M. Neiman

**Affiliations:** Department of Biochemistry and Cell Biology, Stony Brook University, Stony Brook, NY 11794-5215, USA; aaron.neiman@stonybrook.edu

I wish to thank all of the authors who contributed papers to this Special Issue on the Formation and Function of Ascospores. While the process of ascospore formation is most extensively studied in *Saccharomyces cerevisiae*, it is highly conserved throughout the ascomycetes. The work described here touches on many aspects of the process including control of entry, formation and dissolution of the limiting membrane, and construction and function of the spore wall in a variety of ascomycetes.

Ascospore formation is usually linked to meiosis. However, it is not clear if this is the case in *Ashbya gossypii*. Wendland provides a timely review of spore formation in *A. gossypii*, highlighting similarities and the differences with *S. cerevisiae* [1]. The environmental conditions that induce this developmental program vary between fungi. Wasserstrom and Wendland and Jun et al. report on factors controlling entry into the sporulation program in *A. gossypii* and *Aspergillus flavus*, respectively [2,3].

Earlier work has shown that *Schizosaccharomyces pombe* cells undergo ‘virtual nuclear envelope breakdown’ (vNEBD) in Meiosis II, in which nuclear proteins equilibrate into the cytoplasm [4]. Yang et al. show that release of the proteasome subunit Rpn11 via vNEBD is required for proper closure of the forespore membrane (the *S. pombe* name for the prospore membrane) and spore formation [5]. In *S. cerevisiae*, Durant et al. demonstrate that the transient localization of the MAP kinase Smk1 to the lip of the prospore membrane is required for proper membrane closure and spore morphogenesis [6]. Capture of the nucleus by a prospore (or forespore) membrane results in cells that are surrounded by two membranes; the spore plasma membrane closest to the nucleus, and an outer membrane which disappears during the process of spore wall assembly [7]. Zhang et al. identify the first mutants defective in outer membrane lysis and characterize the consequences of this defect in *S. pombe* [8].

The spore wall is the defining feature of the ascospore, and four studies added to our understanding of the organization of this remarkable extracellular matrix. Tahara et al. have used a quick-freeze deep etch electron microscopy technique to reveal the architecture of the surface layers of the *S. pombe* spore wall [9]. The spore wall of *S. cerevisiae* contains an outer layer consisting of a polymer of the di-amino acid dityrosine. Basiony et al. characterize the product of the Dit1 enzyme, responsible for the first step in dityrosine synthesis [10]. In addition to dityrosine, the outer spore wall of *S. cerevisiae* is composed of chitosan and a third, uncharacterized component [11]. Chrissian et al. demonstrate that this third component is a tryglyceride, and that a similar set of constituents—chitosan, neutral lipid, and a polyphenol polymer—are a conserved feature of the melanized cell wall of the basidiomycete *Cryptococcus neoformans* [12]. The ascospores of *Talaromyces macrosporus* are highly stress resistant and will not germinate unless first exposed to extremes of heat or pressure. Dijksterhuis et al. identify a small protein that is released from the spore wall under germination conditions and characterize the phenotype of spores lacking this key spore wall component [13].

The papers of this Special Issue highlight the fascinating cell biology of this highly conserved developmental process. My thanks again to all of the contributors.
